# Evidence against the role of toll-like receptors 7 and 8 in sex selection in mice, cattle, and humans

**DOI:** 10.1016/j.isci.2025.113164

**Published:** 2025-07-18

**Authors:** Ruifeng Zhao, Jiaxi Liu, Azizollah Bakhtari, Jianing Shen, Alexa Fayad-Costa, Bin Wang, Xiuchun Tian

**Affiliations:** 1 Department of Animal Science, University of Connecticut, 1390 Storrs Road, Storrs, CT 06269-4163, USA; 2 Center of Assisted Reproduction, Henan Provincial Hospital, 6 Dongtinghu Road, Zhengzhou, Henan 451162, China

**Keywords:** Natural sciences, Biological sciences, Zoology, Gene products

## Abstract

Recent studies suggested that treating sperm with R848, a ligand for the X-linked Toll-like receptors 7 and 8 (TLR7/8) in mice, goats, and cattle, could selectively reduce the motility of X chromosome bearing sperm (X-sperm). This reduction enables the separation of X- and Y-sperm and thereby sex selection. However, through three species and multiple methods, our results challenged prior published data. We demonstrated that TLR7/8 were equally expressed in the X- and Y-sperm via western blotting (bovine), immunofluorescence staining (mouse, bovine, and human), flow cytometry (mouse, bovine) and *in vitro* embryo production (mouse). Approximately 90% of murine, bovine, and human sperm exhibited positive staining for TLR7/8. Meanwhile, sperm treated with R848 and swim-up did not differ in the X:Y ratio as shown by TaqMan real-time PCR. Taken together, our findings question the restriction of TLR7/8 to the X-sperm and the value of R848 treatment as a viable method for sex selection.

## Highlights

• While X-linked, TLR7/8 proteins were equally present in bovine sorted X- and Y-sperm
• Approximately 90% of mouse, bovine and human sperm stained positively for TLR7/8
• TLR7/8 were present on tails and acrosomes of mouse, bovine, and human sperm
• R848, a TLR7/8 agonist, did not separate X- and Y-sperm

## Graphical abstract

**Figure:**
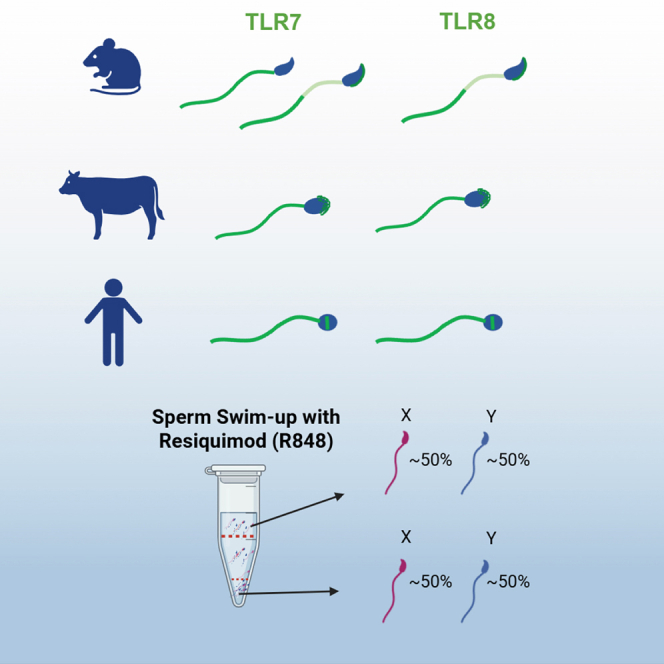

## Introduction

Sex-selection by separating X- and Y chromosome bearing sperm (X- and Y-sperm) is effective and convenient in both humans and farm animals, particularly in dairy cattle.^1^ Many methods have been investigated for separating X- and Y-sperm, but the only reliable and widely applied is the Beltsville method, which uses fluorescence-activated cell sorting (FACS) to detect the DNA content of sperm stained with Hoechst-33342.^2^^,^^3^ The majority of the sperm, however, cannot be assigned to either X- or Y chromosome bearing, resulting in low sperm counts in the commercially sorted semen straws. The method also has the potential to preferentially select for sperm with a smaller Y chromosome, which has already lost most of its DNA during evolution.^4^

Recently, Umehara and colleagues reported that the X- and Y-sperm differentially expressed Toll-like receptor 7 and 8 (TLR7/8) in the mouse and bovine, and that these two X-linked genes were present in ∼50% of the sperm population.^5^^,^^6^ Furthermore, by treating sperm with ligands of TLR7/8, resiquimod (R848) or imiquimod (R837), the nuclear factor κB (NF-κB) and glycogen synthase kinase-3 α/β (GSK3α/β) were phosphorylated, which, in turn, blocked the hexokinase activity, and selectively suppressed the motility of the X-sperm.^5^^,^^6^ Since these initial reports, five primary studies have been published in which separating X- and Y-sperm through TLR7/8 ligand treatments was studied in the mouse, bovine and goat.^7^^,^^8^^,^^9^^,^^10^^,^^11^ For example, Ren et al. reported that approximately 50% of ram sperm exhibited TLR7/8 immunostaining, enabling the separation of X- and Y- sperm through R848 treatment.^7^ Similar findings were shown by Wen et al. in the bovine.^8^ Huang et al. generated a sex ratio bias in goats after combining R848 with an alkaline semen diluent.^9^ Additionally, Hou et al. reported that the use of other TLR7/8 ligands, such as dsRNA-40 and dsRNA-DR, also facilitated the separation of mouse X- and Y-sperm following swim-up.^10^

TLRs belong to the superfamily of transmembrane interleukin-1 receptor (IL-1R) and play critical roles in distinguishing self and foreign in innate immunity.^12^ A total of 13 TLRs have been identified in mammals, with 10 in humans (TLR1-TLR10),^13^ and 12 in mice (TLR1-TLR9, TLR11-TLR13).^14^ TLR3, 7, 8, and 9 are expressed in mouse sperm and have been found to impact sperm motility and fertilization.^13^^,^^15^

Proteins encoded by sex chromosome-linked genes, however, are not automatically candidates to distinguish X- and Y-sperm. This is because male germ cells develop in a syncytium during spermatogenesis and share cellular contents via cytoplasmic bridges.^16^ Disrupting the cytoplasmic bridges before the completion of the first meiotic division caused germ-cell developmental arrest and apoptosis.^17^ Additionally, during meiosis, the X- and Y chromosomes are enclosed in the “sex body”, which inhibits gene expression and further conceal the differences between X- and Y-sperm.^18^ Gene expression of the sex chromosomes continue to be suppressed after meiosis and into spermiation through post-meiotic sex chromatin.^19^ While several proteins including sperm motility kinase 2a/2b (Smok2a/ab), sperm adhesion molecule (Spam1), and sphingomyelin phosphodiesterase 1 (Smpd1) have been explicitly shown to escape sharing between X- and Y-sperm,^20^^,^^21^^,^^22^ these are autosomal genes and TLR7/8 are not among them.

We set out to pursue this chemical sperm separation method for mouse and bovine sex selection. However, we observed contradictory results to those of the aforementioned studies. Using a stringent experimental design, a series of different confirmation methodologies, and three different species, we demonstrated that TLR7/8 were present in most sperm of the mouse, bovine, and human, regardless of their sex chromosome enclosure. R848 treatment, combined with sperm swim-up, was unable to separate X- and Y-sperm.

## Results

### R848 treatment of mouse sperm compromised the development of IVF embryos, yet failed to alter the sex ratio

**Figure 1:**
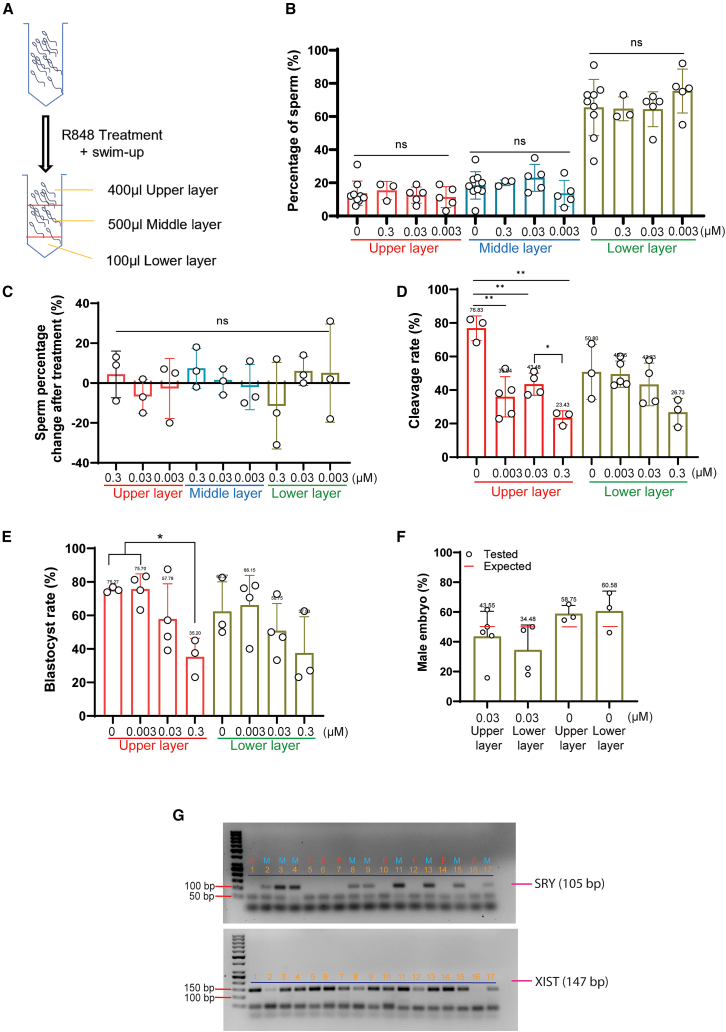
Effects of TLR7/8 ligand (R848) on murine sperm partitioning and *In vitro* fertilization (IVF) (A) The schematic diagram of R848 treatment and swim-up layer assignment. (B) The percentages of sperm in different layers after treatment with R848 at various concentrations, ns = not significant (*p >* 0.05). (C) The changes in the percentages of sperm of the same individual mice before and after R848 treatment in each swim-up layer. Data in (B and C) were analyzed using one-way ANOVA. (D and E) Cleavage (D) and blastocyst (E) rates of embryos derived from R848-treated sperm of different layers. Data were analyzed with one-way ANOVA followed by Tukey’s post-hoc tests. ∗*p* ≤ 0.05, ∗∗*p* ≤ 0.01. (F) The percentages of male embryos derived from IVF using sperm treated with 0 or 0.03 μM R848. The expected male embryo ratio was 50% (red bar). All values are mean ± SD of at least three replicates. (G) Representative agarose gel images of embryo sex determination by PCR. The product sizes for SRY and XIST were 105 and 147 bp, respectively. The bands below the specific ones were likely primer-dimers. Each lane represented an individual blastocyst. Lane marked with “F” and “M” were deemed to be female and male embryos, respectively.

We attempted to replicate the reported observations by treating mouse sperm with 0, 0.003, 0.03, and 0.3 μM R848, the same dose range as in the initial report,^5^ and collected the upper, middle, and lower layers after swim-up (Figure 1A). Unexpectedly, the increasing concentrations of R848 did not alter the proportion of sperm among layers, as opposed to the original report. Specifically, ∼60% of the sperm were located in the lower layer and 20% each in the middle and upper layers (Figure 1B). We also tried to use treated and untreated sperm from the same mouse to eliminate potential washout effects among different mice. We observed insignificant changes in the percentage of sperm in each layer following R848 treatment (Figure 1C). These variations were random and inconsistent across individual mice, representing biological variability. Therefore, increasing concentrations of R848 did not progressively inhibit sperm motility in swim-up. Sperm of both the upper and lower layers after treatment, however, produced lower fertilization and embryo development rates. Specifically, upon treatment with 0.003, 0.03, or 0.3 μM R848, sperm of the upper layer produced dose-related decreases in cleavage rates to 35.94%, 43.48%, and 23.43%, respectively, compared to untreated sperm of the same layer (76.83%; Figure 1D). Sperm of the lower layer after treatment with 0.3 μM R848 showed a decreasing trend in cleavage rate from 50.80% to 26.73% (*p* = 0.09; Figure 1D). Blastocyst development from cleaved embryos reduced significantly from 75.27% to 35.20% in the 0.3 μM R848 upper layer group (Figure 1E).

We then determined the sex ratio of embryos fertilized with sperm treated with 0.03 μM R848, also the same concentration used in the initial report.^5^ While we observed some skewing toward females, this was not restricted to one particular swim-up layer. Overall, there was no significant difference in the sex ratio of fertilized embryos upon any treatment (Figures 1F and 1G). Together, R848 treatment compromised fertilization and embryo development but failed to alter the sex ratio of fertilized embryos.

### X- and Y-sperm could not be separated by R848 treatment

**Figure 2:**
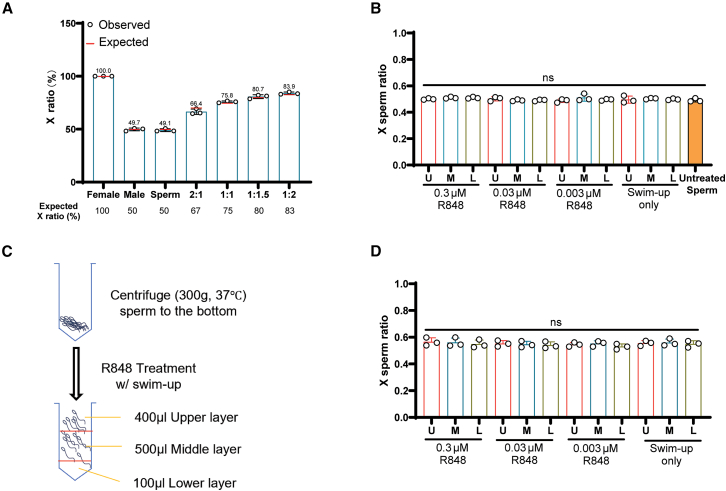
TLR7/8 ligand (R848) treatment did not separate X- and Y- sperm (A) Validation of TaqMan real-time PCR for sex chromosome ratio determination by using different ratios of male vs. female mouse genomic DNA. The expected ratios are marked with red bars. Data were analyzed with one sample t test. (B) TaqMan real-time PCR determination of the X-sperm ratios in different layers of sperm treated with R848. Data were analyzed with one-way ANOVA, ns = not significant (*p* > 0.05). (C) Schematic diagram of centrifugation of sperm before R848 treatment and swim-up. (D) TaqMan real-time PCR determination of the X-sperm ratios in different layers of sperm from (C). Data were analyzed with one-way ANOVA. All values are mean ± SD from at least three replicates.

Because our embryo sex ratio results were different from those of prior studies, we questioned whether this method could separate X- and Y-sperm by selectively inhibiting the motility of the X-sperm in the swim-up assay. We therefore determined the percentages of X- and Y-sperm in each layer by the absolute quantitative TaqMan real-time PCR. To validate the quantification accuracy, we mixed genomic DNA from male and female mice at ratios of 2:1, 1:1, 1:1.5, and 1:2. We obtained X:Y ratios closely resembling those of the expected (Figure 2A). We then determined the percentage of X-sperm in each layer after R848 treatment. We found that regardless of the concentration of R848, the percentage of X-sperm remained close to 50% in all layers (*p* = 0.45; Figure 2B), indicating no selective mobility inhibition.

While observing sperm under the microscope, we saw immobilized sperm in the upper layer and wondered if motile sperm prevented immobilized sperm from settling to the lower layer, thus compromising the test results. While the methods in original or subsequent studies did not describe centrifugation of sperm before swim-up, we centrifuged the sperm at a low speed (300 g) at 37°C before swim-up to bring all sperm to the bottom (Figure 2C). This should allow only motile sperm to swim up and be present in the upper layer. However, we did not see a difference in the percentage of X-sperm in any layer after any level of treatment (*p* = 0.91) (Figure 2D). Interestingly, we observed a slightly skewed percentage toward X-sperm in all layers by ∼5%. This may be attributed to the reported greater fragility of Y-sperm than X-sperm.^23^ It is possible that during the additional centrifugation, some Y-sperm did not survive or became immobilized. These sperm then precipitated to the bottom and were excluded from layer collection. In summary, we found that R848 could not differentially affect the motility of mouse X- or Y-sperm in swim-up.

### Approximately 90% of murine sperm stained positively for TLR7/8

**Figure 3:**
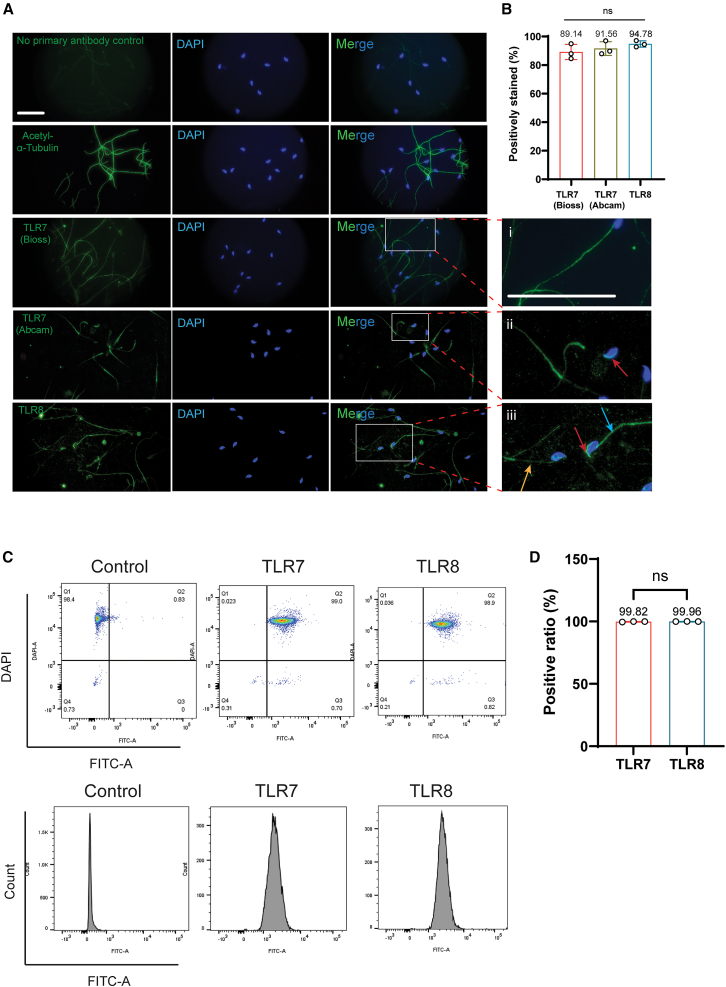
Localization of TLR7/8 in murine sperm (A) Immunofluorescence staining of TLR7/8 (green), acetyl α-tubulin (green), and DNA (blue) in murine caudal epididymal sperm. (i) The Bioss antibody against TLR7 stained the whole sperm tails; (ii) The Abcam antibody against TLR7 stained the lower half of the sperm tail as well as the acrosome of the sperm (red arrow); (iii) TLR8 antibody stained either the lower half (yellow arrow) or the entire sperm tail (blue arrow), as well as the acrosome (red arrow). Scale bar, 25 μm. (B) The percentages of murine sperm stained positively for TLR7/8 by immunofluorescence. (C) Flow cytometry analysis of mouse sperm stained with TLR7/8 and DAPI. In controls (left panels) the sperm were probed with the secondary antibody only and provided the cutoff (vertical lines in the upper panels) for stained and unstained sperm. The cells were detected by the FITC-A (*x* axis) and DAPI (*y* axis) channels. Each dot represents one sperm. The upper panel showed the distribution of the positively and negatively stained sperm, the lower panel displayed the intensity of fluorescence of the sperm (*x* axis) and sperm counts (*y* axis). The middle and right panels represent sperm stained for TLR7 and TLR8, respectively. Nearly all sperm were stained by both antibodies. (D) The percentages of mouse sperm positively stained for TLR7 or 8 as detected by flow cytometry. All values are mean ± SD of at least three replicates. Data were analyzed using t tests. ns = not significant (*p* > 0.05).

Due to the presence of cytoplasmic bridges, sex bodies, and post-meiotic sex chromatin during spermatogenesis, we wondered whether the X-linked TLR7 and/or TLR8 were restricted to or were primarily present in X-sperm only. Firstly, we performed immunofluorescence staining of the mouse sperm (Figure 3A). Using the same antibody (Bioss, bs-6601) and dilution ratio as reported in the original report and subsequent protocol,^5^^,^^6^ we only found weak signals of both TLR7 and TLR8 compared to those of acetyl-α-tubulin (control). We believe this observation is consistent with the report by Navarro-Costa et al.^24^ who found extremely low levels of TLR7/8 transcription in mouse sperm. By titrating the dilution ratio (from 1:100 to 1:10) of the primary antibody, we gradually increased the visibility of TLR7/8 signals (Figure S1). We found that for TLR7 the entire sperm tails were stained, agreeing with Umehara et al.^5^ However, 89.14% of all mouse sperm in our study were positively stained (Figure 3B), as opposed to the ∼48% in the prior report.^5^ This surprising difference prompted us to use a different TLR7 antibody from another supplier (Abcam, ab24184) to further validate our results (Figure 3A). This antibody stained the lower portion of the sperm tail, and the acrosomal region of the sperm heads (Figures 3Ai–3Aii). The staining of the acrosome is logical because the TLRs have been located in the Golgi apparatus, which becomes the acrosome in sperm.^25^^,^^26^ Regardless of the staining location, the percentages of TLR7-stained sperm by either antibody were close to 90% (Figure 3B).

For TLR8 two tail staining patterns were observed, some were stained for the entire tails while others the lower portion of the tails (Figure 3Aiii). Combined, 94.78% of the sperm were positively stained (Figure 3B). To demonstrate that these observations were not unique to one mouse strain, we used the sperm of another mouse strain (C57BL/6) and obtained the same observations (Figure S2). Because some of our staining patterns and all percentages were different from those of the original report^5^; we conducted flow cytometry to analyze more sperm simultaneously. Consistent with manual counting, nearly 100% sperm were positive for both TLR7/8, while nearly 100% of the sperm stained with the fluorescence-labeled secondary antibody (negative controls) had no signals (Figures 3C and 3D).

### Both sorted bovine X- and Y-sperm expressed TLR7/8

**Figure 4:**
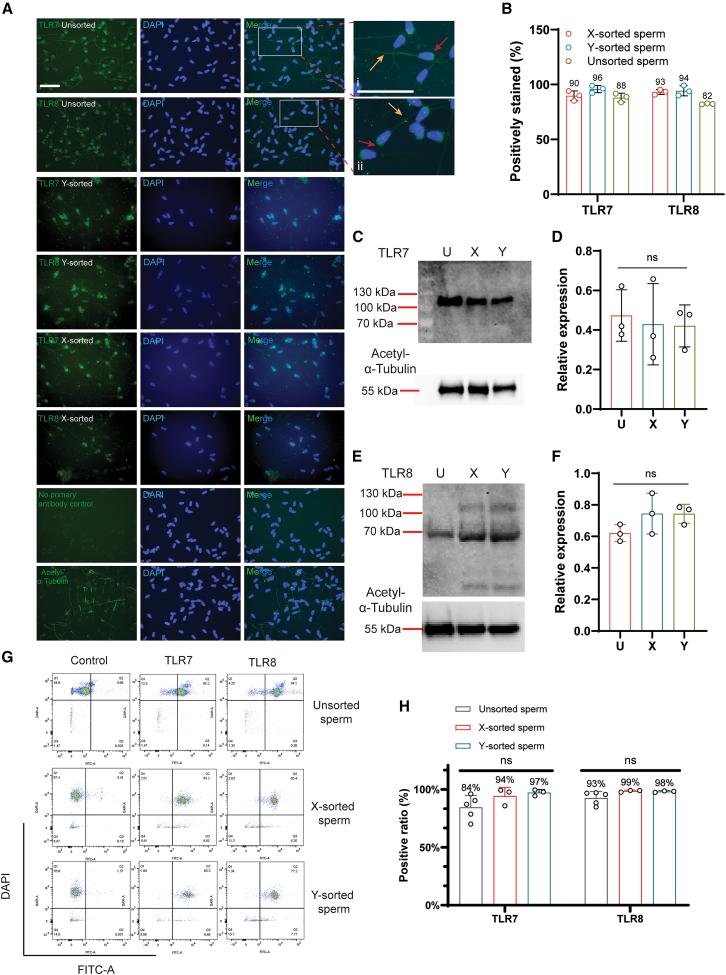
Detection of TLR7/8 on bovine X- and Y-sorted sperm (A) Immunofluorescence of TLR7 (green), TLR8 (green), acetyl α-tubulin (green) and DAPI (blue) on frozen-thawed un-sorted, X- and Y-sorted bovine sperm. (i–ii) Both TLR7 and TLR8 were stained at the acrosomal regions of the bovine sperm heads (red arrows), and tails (yellow arrows). Scale bar, 21 μm. (B, C, and E) (B) The percentages of positively stained bovine sperm for TLR7 and TLR8. Western blot analyses of TLR7 (C) and TLR8 (E) on bovine un-sorted (U), X-sorted (X) and Y-sorted (Y) sperm. TLR7 and TLR8 were resolved and blotted using non-reduced and reduced samples, respectively. (D and F) Quantification of the TLR7 (D) and TLR8 (F) bands from western blots. Data were analyzed using one-way ANOVA, ns = not significant (*p* > 0.05). (G) Flow cytometry analysis of bovine sperm stained for TLR7/8 and DAPI. In controls (left panels) the sperm were probed with the secondary antibody only, which provided the cutoff (vertical line) for stained and unstained sperm. The cells were detected by the FITC-A (*x* axis) and DAPI (*y* axis) channels. Each dot represents one sperm. The middle and right panels represent sperm stained for TLR7 and TLR8, respectively. Nearly all sperm were stained by both antibodies. (H) The percentages of positively stained bovine sperm as detected by flow cytometry. Data were analyzed using one-way ANOVA. All values are mean ± SD of at least three replicates, ns = not significant (*p* > 0.05).

The prior results that only ∼50% mouse and bovine sperm positively stained for TLR7/8 by immunofluorescence^5^^,^^6^ were not only inconsistent with our mouse results, but also with multiple prior proteomic reports which did not identify TLR7 or TLR8 as differentially expressed between bovine sorted X- and Y-sperm.^27^ We therefore incorporated commercial, unsorted, and sorted bovine X- and Y-sperm (>90% sorting accuracy) to further investigate TLR7/8 expression patterns.^28^ Similar to the mouse, bovine sperm showed TLR7/8 positive staining on the acrosomes and tails of both X- and Y-sorted sperm (Figures 4Ai and 4Aii). Notably, we only observed 4%–12% negatively stained sperm. However, the percentages of negative staining were not significantly different among unsorted, X-, or Y-sorted sperm (Figure 4B). We again conducted flow cytometry of bovine sperm to further confirm the manual quantification results. Over 90% of bovine sperm were positively stained for both TLR7 and TLR8 in all three types of sperm (Figures 4G and 4H). We also conducted western blotting for TLR7/8 using unsorted, X-, and Y-sorted sperm. TLR7 and TLR8 were detected in all three groups at the expected sizes of ∼120 kDa (uncleaved) and ∼70 kDa (cleaved from the reduced condition used), respectively (Figures 4C and 4E). No significant differences in the relative protein levels of either TLR7 or TLR8 were found between the sorted sperm groups (Figures 4D and 4F). Therefore, we conclude that TLR7/8 proteins were not only present in both bovine X- and Y-sperm but were also distributed at equal levels. These data are in agreement with prior published proteomics results of the bovine sorted sperm.^29^^,^^30^^,^^31^

### The expression of TLR7/8 in human sperm was not related to the sex chromosome

**Figure 5:**
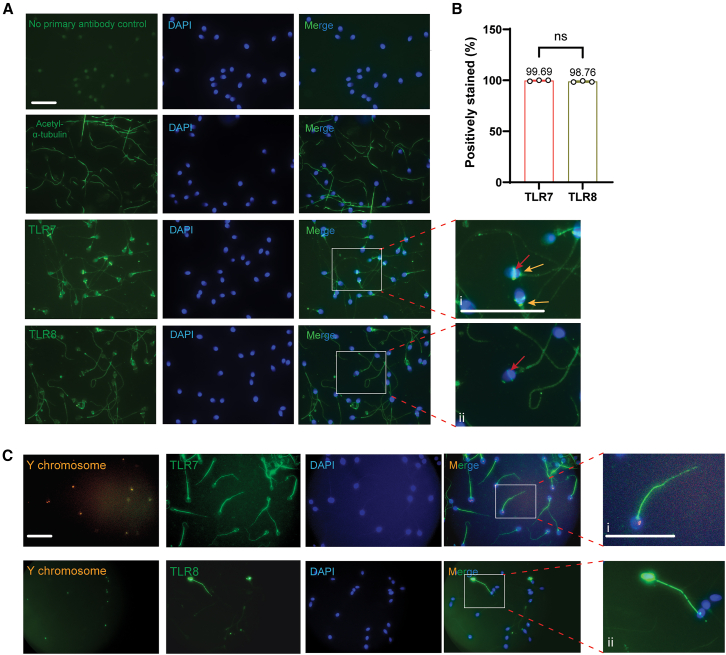
Localization of TLR7 and TLR8 on human sperm (A) Representative images of immunofluorescence of TLR7/8 (green) and DAPI (blue) on human sperm. (i–ii) Both TLR7 and TLR8 stained human sperm tails as well as the equator regions of the heads (acrosomes, red arrows). TLR7 was also seen in the head-tail connecting apparatus (Yellow arrow). Scale bar, 22 μm. (B) The percentages of positively stained human sperm by TLR7/8 immunofluorescence. All values are mean ± SD of at least three replicates. Data were analyzed using t tests, ns = not significant (*p* > 0.05). (C) Y chromosome fluorescence *in situ* hybridization (FISH, orange) and immunofluorescence for TLR7 and TLR8 (green) in a human sperm donor because his staining patterns and percentages were different from other donors. The inset (i) shows the details of TLR7 tail stain and the Y chromosome signal. TLR8 stained few sperm and the stain was seen on the entire sperm (ii). Immunofluorescence staining preceded FISH. Scale bar, 25 μm.

We expanded our efforts to determine if the results of the mouse and bovine would also be true in humans. Unsurprisingly, 99.69% and 98.76% of human sperm stained positively for TLR7 and TLR8, respectively (Figures 5A and 5B). The immunofluorescence signals for TLR7/8 were localized on both the tail, head-tail connecting apparatus at the neck region, and equator of the sperm head which is also part of the human acrosome (Figures 5Ai and 5Aii).^32^ Interestingly, sperm from one donor presented a different staining pattern. TLR7 was only found on the sperm tail (Figure 5Ci). TLR8, however, was observed over the entire sperm (Figure 5Cii). Quantitatively, 75.00% of this donor’s sperm were positive for TLR7 (Table 1), but only 6.56% stained positively for TLR8 (Table 2), possibly due to low expression levels. Because this donor’s staining patterns and percentages of positively stained sperm were different from other donors, we added fluorescence *in situ* hybridization (FISH) of the Y chromosome to sperm already stained for TLR7 or TLR8 (Figure 5C). Of those positively stained for TLR7, 50% were also positive for the Y chromosome, suggesting that TLR7 was present in both X- and Y-sperm (Table 1). For TLR8 positive cells, however, as high as 75% were Y-sperm, again demonstrating that the presence of TLR8 was not restricted to the X-sperm (Table 2).

**Table 1:** TLR7 immunostaining and fluorescence *in situ* hybridization (FISH) of the Y chromosome in the sperm of a human donor

Signal combinations	Sperm counted	Expected ratio^a^	Observed ratio	Binomial proportional confidence interval
DAPI	224	100%	100%	–
Y+	112	50%	50.00%	[43.45%, 56.55%]
Y-	112	50%	50.00%	[43.45%, 56.55%]
TLR7+	168	50%	75.00%^b^	[69.33%, 80.67%]
TLR7+/Y+	96	0%	57.14%^b^	[49.66%, 63.62%]
TLR7+/Y-	72	100%	42.86%^b^	[36.38%, 50.34%]
TLR7-/Y+	16	100%	28.57%^b^	[16.74%, 40.40%]
TLR7-/Y-	40	0%	71.43%^b^	[59.60%, 83.26%]

a The expected ratios for all TLR7 counts were calculated based on the assumption that the protein was restricted to X-sperm only.

b The observed ratios significantly deviated from the expected ratios because the latter fell outside the binomial proportional confidence intervals calculated for the observed ratios.

**Table 2:** TLR8 immunostaining and FISH of the Y chromosome in the sperm of a human donor

Signal combinations	Sperm counted	Expected ratio^a^	Observed ratio	Binomial proportional confidence interval
DAPI	122	100%	100%	–
Y+	62	50%	50.82%	[41.95%, 59.69%]
Y-	60	50%	49.18%	[40.31%, 58.05%]
TLR8+	8	50%	6.56%^b^	[2.17%, 10.95%]
TLR8+/Y+	6	0%	75.00%^b^	[44.99%, 105.01%]
TLR8+/Y-	2	100%	25.00%^b^	[0%, 55.01%]
TLR8-/Y+	56	100%	49.12%^b^	[40.02%, 58.22%]
TLR8-/Y-	60	0%	51.72%^b^	[42.63%, 60.81%]

a The expected ratios for all TLR8 counts were calculated based on the assumption that the protein was restricted to X-sperm only.

b The observed ratios significantly deviated from the expected ratios because the latter fell outside the binomial proportional confidence intervals calculated for the observed ratios.

Together, our results demonstrated that the X-linked TLR7/8 proteins were present in the majority of the mouse, bovine, and human sperm, and their distribution was not restricted to the X-sperm. Treating sperm with TLR7/8 ligand did not generate X- and Y-sperm partitioning or skewed sex ratio after fertilization.

## Discussion

Using sperm from the mouse, bovine, and human and different methods for reaffirmation, we demonstrated that while the TLR7/8 genes are X-linked, their gene products are equally present on both the X- and Y-sperm, thus reflecting complete sharing of these two proteins between the two types of haploid mature gametes. Prior to the present study, only a few genes have been explicitly shown to undergo such sharing. These are the X-linked A-kinase anchoring protein 4,^33^ the autosome-encoded protamines 1 and 2,^33^^,^^34^ and two reporter transgenes under the control of the protamine 1 promoter.^35^^,^^36^ Our data here brought the number of deliberately characterized and completely shared X-linked genes from the previously reported one to three.^24^ Additionally, we evinced that treatment of sperm with TLR7/8 ligand (R848) did not separate X- and Y-sperm based on their sex chromosome enclosure and, therefore, cannot be used for sex preselection. Together, our results provided further evidence that the successful maintenance of a balanced sex ratio across generations of mammalian reproduction involves complex and redundant mechanisms including sharing of X-linked gene products and in some cases the expression of the inactive form of such gene-products.

Our results contrast to those of prior studies and are attributable to a number of factors. First, the staining patterns of TLR7/8 were inadequately reported in all three previously studied species, mice, goats, and cattle.^5^^,^^6^^,^^7^ For example, in the mouse, only sperm tail staining was described for both TLR7 and TLR8.^5^ While we found mouse sperm were not only positively stained for TLR7/8 in the tails but also at the acrosome. In fact, in the subsequent publication by the same authors^6^ an image in Figure 4 showed that the mouse acrosomes were obviously and strongly stained for TLR7, yet these sperm were still called negatively stained, and the acrosome stain was unmentioned. Similarly, the obvious TLR7/8 stain on acrosomes and TLR8 stain at the head-tail connecting apparatus was also unnoted in the report of goat sperm.^7^ In bovine sperm, we again found tail as well as acrosome stains. In the two prior bovine studies,^6^^,^^8^ the obvious positive TLR7 stains at the acrosome and head-tail connecting apparatus were overlooked in one study.^6^ While in the other study the “cranial stain” for TLR7 and acrosome stain as well as the “connecting piece” for TLR8 were noted,^8^ the image (Figure 3A, upper middle panel) showed that the entire heads of ejaculated sperm were stained for TLR7. The same study also stained bovine seminiferous tubules. TLR7 could be seen to strongly stain nearly all spermatogonia^8^ (Figure 1A), pointing to the possibility that TLR7 could be passed down to both X- and Y-spermatids. Yet this important staining pattern was unmentioned. Additionally, the low-resolution image^8^ (Figure 1B) showed indiscernible stains for TLR7/8 on spermatids and the description for counting of the stained cells was very vague in the figure legend. Yet the ∼40% of TLR7^+^/8^+^ cells was nonetheless resulted.^8^

Our consistent finding of TLR7/8 stains on acrosomes of mouse, bovine, and human sperm extended prior reported localizations for these two proteins. In somatic cells, TLR7/8 are known to localize in endosomes, which is likely related to their autophagia activities.^25^ Acrosomes of the sperm are derived from the Golgi apparatus,^37^ which is involved in the recycling of late endosomes and are therefore logical locations to find TLR7/8 stains.

Second, while we determinately showed that TLR7/8 were present in both X- and Y-sperm of all three species, prior studies did not convincingly demonstrate that TLR7/8 were only present in the X-sperm. For example, in Figure S3^5^ mouse sperm were stained with TLR7, DAPI, and X chromosome painting in an attempt to prove the co-localization of the X chromosome and TLR7. However, among the 16 sperm shown, only five had positive X chromosome paint, of which four had more than one punctate spot, pointing to a procedural problem. In the same figure, TLR7 only stained three of the 16 sperm, and two X-positive sperm were negative for TLR7. These images could not confirm the conclusion reached, which was “TLR7/8 were localized in only X-spermatids”.^5^ Furthermore, when using flow cytometry to analyze TLR7/8-stained sperm, a negative control was absent.^5^^,^^6^ Without such a control, the reported “two peaks” could be high and low signal intensities instead of the assumed positive and negative staining.^5^^,^^6^ In contrast, we first established a negative control that included only the secondary antibody to establish a baseline. This was then used as a cutoff to distinguish negatively and positively stained cells. We found that over 90% of sperm stained above the negative cutoff in both mouse and bovine samples, convincingly demonstrated that TLR7/8 were not restricted to the X-sperm only.

Third, a different flow cytometry analysis based on Hoechst 33342 staining was previously attempted on caprine and bovine sperm of the upper and lower layers after R848 treatment.^7^^,^^8^ In goats, two populations of un-revealed Hoechst 33342 staining intensity were shown for the control sperm and were assumed to be X- and Y chromosome bearing^7^ (Figure 5E left panel) without validation on their sex chromosome enclosure. For R848 treated sperm, the upper and lower layers were analyzed in two separate plots, each contained a major and a minor peak^7^ (Figure 5E middle and right panels). Again, these peaks were assumed to be the separated X- and Y-sperm without validation. Interestingly, these two plots^7^ (Figure 5E middle and right panels) showed that the presumed Y- and X-sperm had the same Hoechst staining intensity, averaged at ∼10^4^, despite of their DNA content differences. This single staining intensity for both X- and Y-sperm is in direct conflict with the data presented in the control plot, which showed their different staining intensities even though the intensities were not shown. In the bovine, similar results and interpretation were reported using the same flow cytometry analyses, also without PCR validation^8^ (Figure 6B in Ref.^8^). The presumed Y-sperm in the control plot, the upper layer presumed Y-sperm in a second plot, and the lower layer presumed X-sperm in a third plot were all shown to have the same staining intensity, averaged again at ∼10^4^. These data are puzzling because the X-sperm in the control plot clearly had higher staining intensity, averaged between ∼10^4^ and 10^5^.

Fourth, while all prior studies concluded/accepted that R848 selectively deterred the motility of the X-sperm because of the assumed restriction of TLR7/8 to the X-sperm, none determined the TLR7/8 protein levels in sperm of the upper and lower layers. The bovine sorted X- and Y-sperm are commercially available and represent great resources for confirmation. Using western blotting we determined that these two proteins were present equally in both bovine X- and Y-sperm. Furthermore, we also showed that at least the TLR7 protein was present in the high molecular weight and un-cleaved form, suggesting that it is inactive in ejaculated sperm.^38^ Although western blots for TLR7/8 were conducted in mouse sperm in the initial study (Figure 1B),^5^ only untreated sperm were used and the molecular weight information was not provided. The authors compared the upper and lower layers for their activities of NF-κB and GSK3α/β by western blotting (Figure 4G), but the same comparison for TLR7/8 was absent.^5^

Fifth, since the initial publication,^5^ all subsequent primary publications, totaling five to date, reported confirmation/acceptance of the initial findings, despite the conflicting data within and among studies detailed previously. Interestingly, a recent report claiming to accept the original findings, presented data that showed otherwise. Yotov et al.^11^ were unable to find significant DNA content difference between the upper- and lower-layer goat sperm after R848 treatment, while a BSA treatment produced significant differences. Additionally, although R848 was supposed to selectively reduce the motility of X-sperm, it was in fact the presumed Y-sperm whose total motility was significantly lowered by the treatment compared to controls (∼85% vs. 100%). The report did not directly compare the motilities between the assumed X- vs. Y-sperm, but the figure showed that their motilities were ∼85% and ∼82%, respectively, of the untreated sperm, unlikely to be significantly different, and again disproving that R848 selectively inhibited X-sperm. Furthermore, Yotov et al.^11^ showed that the percentages of fast, medium progressive motile spermatozoa, and non-progressive sperm were indifferent between the assumed X- and Y-fractions. Therefore, the close examination of data by Yotov et al.^11^ pointed to the invalidation of the R848 method. Of note, this is not the first report in which sperm motility change was not what was expected. It was documented^7^ that the motility, average path velocity, average straight-line velocity, and average curve line velocity of R848-treated goat sperm only reduced by a few percentage points compared to controls (Figures 4D–4G). If nearly 50% of the treated sperm (i.e., X chromosome bearing) suffered from impaired motility, a much greater percentage reduction in all motility parameters is expected.

Sixth, while a unique aspect of four prior studies was the breeding outcome, major discrepancies were reported among them. Umehara et al.^5^ documented the highest success with a sex ratio bias of 81%–83%. Ren et al.^7^ obtained a similarly highly skewed ratio of 8:1 (88.89%) in goats, but only nine embryos from two does were studied. All subsequent breeding sex ratio biases were much smaller. Huang et al.^9^ added an X-sperm enrichment measure by combining a weak alkaline incubation (pH = 7.4) with R848 treatment, but the female offspring rate was only 62.79% from a large artificial insemination trial (378 kids from 251 does) using the lower layer of the expected X-sperm. In comparison, the untreated control sperm generated 47.65% females from a total of 405 kids and 237 does. Unfortunately, the study did not include an alkaline only treatment group, making it hard to determine if the skewed sex ratio was an effect from alkaline or R848 ^9^. Yet, the same alkaline treatment had been studied previously and reported to produce a biased IVF embryo sex ratio of 70.27% vs. the control at 51.43%.^39^ Together, the breeding results of the two studies suggest that R848 did not generate more sex ratio bias in goats than the alkaline treatment itself.

Seventh, the most puzzling aspect of all prior R848 sexing results was the inconsistency with the well-known and special features of spermatogenesis. Cytoplasmic bridges among male germ cells lead to sharing of contents between X- and Y-spermatids, sex bodies inhibit gene expression of the sex chromosomes during meiosis, and both the X- and Y chromosomes continue to be compartmentalized post-meiosis, and their expression is inhibited throughout spermiogenesis into mature sperm. These evolutionarily conserved structures/mechanisms conceal differences between X- and Y-sperm and serve to ensure that sex ratio approximates 1:1 in natural reproduction. However, only cytoplasmic bridges were lightly discussed in three of the seven prior publications.^5^^,^^6^^,^^9^ With no supporting evidence, it was speculated that “TLR7/8 are expressed at the latter stage of spermiogenesis”^5^ and after “closure of the intercellular bridges”.^6^ However, there have been no reports demonstrating that cytoplasmic bridges indeed close during spermiogenesis. The prevailing model is that when mature sperm are released, the residual bodies are still connected.^40^ Furthermore, Wen et al.^8^ staining of the bovine seminiferous tubules clearly revealed that the spermatogonia were more strongly stained than spermatids or sperm, an experimental observation conflicting with the aforementioned speculation, and suggested that at least some TLR7 were “inherited” by both the X- and Y-spermatids. While it was argued^9^ that only 28% of small granules entering the cytoplasmic bridges were successfully transported to the other cells as shown by Ventela et al.^41^ and that unequal distribution of X-linked TLR7/8 was therefore possible, the specific, unevenly distributed mRNAs or proteins were not studied by Ventela et al. Such detailed information can be gleaned from a few additional sources. Bhutani et al.^42^ reported that 31%–52% of mouse, bovine, and human genes were differentially expressed between the two alleles in spermatids and were termed genoinformative markers, suggesting wide-spread incomplete mRNA sharing. However, most of these genoinformative markers were located in autosomes or 3′ untranslated regions. Only 104 and 46 genoinformative markers were X- and Y-linked, respectively, and 43% of them were predicted genes. These data in fact indicate abundant sharing of the known X- and Y-linked genes. More important to the present discussion, neither TLR7 nor 8 was among the X- or Y-linked genoinformative markers. Furthermore, Namekawa et al.^19^ showed that while post-meiotic sex chromatin continued to inhibit gene expression of the X- and Y chromosomes in spermatids and mature sperm, 13% of X-linked genes were not inhibited post-meiotically. Yet, TLR7/8 were again not among the un-inhibited genes, again contradicting the aforementioned speculation that “TLR7/8 are expressed at the latter stage of spermiogenesis”. We must point out that the lack of mRNA sharing does not translate to the lack of protein sharing. Direct measurements of the proteins themselves are the best approach to answer the TLR7/8 localization question. Prior proteomics studies have identified approximately 40 differentially expressed proteins between sorted bovine X- and Y-sperm^29^^,^^30^^,^^31^ and TLR7/8 were once more missing from the differential list. In fact, the products of only three autosomal genes, Smok2a/2b, Spam1, and Smpd1, were explicitly studied to date at the protein levels or via functional assays and shown to escape sharing between spermatocytes.^20^^,^^21^^,^^22^ Our immunostaining and flow cytometry data from the mouse, bovine, and human strongly suggest that the X-link TLR7/8 are shared between X- and Y-sperm, and our western blotting data from the bovine demonstrated complete sharing of these two proteins. Together with the observations that both TLR7/8 were lowly expressed in sperm and TLR7 was present in the inactive form, the validity of the R848 sperm separation method is at least questionable.

Eighth, intrigued by the reported R848 effects, double-stranded RNA-40 (dsRNA-40) and RNA-DR (dsRNA-DR) as alternative TLR7/8 ligands were used to treat goat sperm and a biased sex ratio of 65.9%–74.93% was obtained in the fertilized embryos.^10^ However, the study did not investigate whether or not TLR7/8 were in the active RNA binding form or if they were differentially present between the X- or Y-sperm. If the expression pattern of TLR7/8 in goats and bovine is evolutionarily conserved, the mechanisms for dsRNA-40 and dsRNA-DR need further investigation. We speculate that the restricted distribution of active TLR7/8 to only X-sperm is not evolutionarily plausible. Single-stranded RNAs (ssRNAs) serve as the most common ligands for TLR7/8.^43^ As many as 27 viruses, including ssRNA virus such as SARS-CoV-2, have been found in human semen.^44^ In bovine semen, ssRNA viruses such as the Schmallenberg virus have also been reported.^45^ These findings suggest that semen can be frequently infected with ssRNA viruses. If TLR7/8 were only present in the X-sperm, any presence of ssRNA virus would cause a disparity in the motility between X- and Y-sperm, and likely a skewed sex ratio downstream. Such a mechanism would produce a detrimental consequence across multiple generations. Corroborating with our postulation and conflicting with RNA-ligand results,^10^ Mihara et al.^15^ observed that culturing mouse sperm with ssRNA did not reduce sperm motility, providing supporting evidence that TLR7/8-related sex selection likely does not work.

Lastly, we found that the TLR7/8 ligand R848 did not alter the distribution of sperm across different swim-up layers, and Yotov et al.^11^ observed that the motility of all sperm was slightly affected. Curiously, however, prior studies either directly stated that R848 treatment induced “upward or downward swimming sperm”,^8^ “swim-up and swim-down sperm”,^5^ or implied the same in a schematic figure^7^ that R848’s effect was to cause Y-sperm to swim upward, yet simultaneously the X-sperm to swim downward. Although these could be simply description errors, such differential effects on X- and Y-sperm are nonetheless in direct conflict with the proposed mechanism that only the X-sperm were selectively inhibited by R848 in motility. The direction of sperm movements was not part of the hypothesis.

### Limitations of the study

Our study is limited on the sperm’s TLR7/8 expression and R848’s effect on sex ratio. We did not study the other functions of R848 and TLR7/8, such as R848’s immuno-stimulation and antiviral properties through TLR7/8, or TRL7/8’s involvement in autoimmune disease inhibition, antiviral, and antitumor effects. However, these effects may not be related to sperm’s motility or fertilization capacity. Additionally, we only counted sperm in different swim-up fractions after R848 treatment and did not measure the sperm’s survival rates or movement speeds. However, we believe counting fractioned sperm is the most biologically relevant and appropriate method here because when applying the sexing method in fertilization, the whole fractions would be used, and data on sperm’s specific movement speeds do not apply. Regardless of the limitations, the observation and conclusion that R848 does not separate X- and Y-sperm still hold true.

## Resource availability

### Lead contact

Information and requests for resources and reagents should be directed to and will be fulfilled by the lead contact, Dr. Xiuchun (Cindy) Tian (xiuchun.tian@uconn.edu).

### Materials availability

N/A.

### Data and code availability

• All data reported in this paper is available from the lead contact upon request.
• This paper does not report original code.
• Any additional information required to reanalyze the data reported in this paper is available from the lead contact upon request.

## Acknowledgments

We thank the sperm donors for providing consents to the study; nurses Yameng Ji and Ge Zhang, and Dr. Ge Bai from the Center of Assisted Reproduction in the Henan Provincial Hospital for assisting the human sperm work; and Dr. Maria Gracia Gervasi, Dr. Yue Su, Dr. Saurav Ranjitkar, and Dr. Jing Jin for providing invaluable advice and discussion. We gratefully acknowledge Dr. Timothy Moore and Dr. Eric Bae of UConn’s Statistical Consulting Services for their support with data analysis. This study was supported by 10.13039/100000199USDA grants 58-8042-5-047 and W4171/5171 and internal funding from the Center of Assisted Reproduction at Henan Provincial Hospital.

## Author contributions

Conceptualization: R.Z., B.W., and X.T.; experimental execution: R.Z., J.L., A.B., and A.F.-C.; methodology: R.Z., B.W., and X.T.; data analysis: R.Z., J.S., B.W., and X.T.; original draft: R.Z. and X.T.; review and editing: R.Z., J.L., A.B., B.W., and X.T.; funding: X.T. and B.W.

## Declaration of interests

The authors declare no competing interests.

## STAR★Methods

### Key resources table

**Table:** 

REAGENT or RESOURCE	SOURCE	IDENTIFIER
**Antibodies**		

TLR7 polyclonal antibody	Bioss	Cat# bs-6601R; RRID:AB_11090775
Anti-TLR7 antibody	Abcam	Cat# ab24184; RRID:AB_447915
Anti-TLR8 antibody	Abcam	Cat# ab180610
TLR8 polyclonal antibody	Invitrogen	Cat# PA5-102413; RRID:AB_2851818
Acetyl-alpha tubulin (Lys40) polyclonal antibody	Invitrogen	Cat# PA5-105102; RRID:AB_2816575
Goat anti-rabbit IgG (H + L) secondary antibody, HRP	Invitrogen	Cat# 32460; RRID:AB_1185567
Goat anti-rabbit IgG H&L (FITC)	Abcam	Cat# ab6717; RRID:AB_955238

**Biological samples**		

Frozen bovine semen	Genex	
Frozen human semen	Henan Provincial Hospital	

**Chemicals, peptides, and recombinant proteins**		

Pregnant Mare Serum Gonadotropin (PMSG)	Sigma	Cat# G4877
human Chorionic Gonadotropin (hCG)	Sigma	Cat# C1063
Human Tubal Fluid (HTF)	Millipore	Cat# MR-070
Resiquimod (R-848)	Novus Biologicals	Cat# NBP2-26231-5mg
Prolong™ Gold anti-fading solution	Invitrogen	Cat# P10144
Isothermal denaturing reagent	Cellay	Cat# SL20041-0500
Antifade+DAPI	Cellay	Cat# SL20013-0001
Proteinase K	Thermo Scientific	Cat# EO0491
QIAamp® DNA Mini Kit	Qiagen	Cat# 56304
FastStart TaqMan® Probe Master	Roche	Cat# 04 673 417 001
RIPA lysis	Thermo Scientific	Cat# 89900
4x NuPAGE LDS Sample Buffer	Invitrogen	Cat# NP0007
Blotting Grade Blocker Non-Fat Dry Milk	Bio-Rad	Cat# 1706404XTU

**Experimental models: Organisms/strains**		

Mouse: C67BL/6	Charles River	
Mouse: Kunming White	Henan Provincial Hospital	

**Oligonucleotides**		

Oligo Y chromosome FISH Probes	Cellay	Cat# OF2-0204-0100
TaqMan real-time PCR probe: SRY: GCAGAATCCCAGCATGCAAA	Sigma	
TaqMan real-time PCR probe: XIST: GATAGACGAACCAGCTCCCA	Sigma	
TaqMan real-time PCR primer: SRY Forward: GTGACACTTTAGCCCTCCGA	Sigma	
TaqMan real-time PCR primer: SRY Reverse: CCACCTGCATCCCAGC TGCTTGC	Sigma	
TaqMan real-time PCR primer: XIST Forward: CCGCCGAGTACTTAGGTCTT	Sigma	
TaqMan real-time PCR primer: XIST Reverse: ACTGCGCGCAGCAACATGCA	Sigma	
PCR primer: SRY Forward: AAGCGCCCCATGAATGCATT	Sigma	
PCR primer: SRY Reverse: TCCCAGCTGCTTGCTGATCT	Sigma	
PCR primer: XIST Forward: CAGAGGAAGAGGAAGGCACG	Sigma	
PCR primer: XIST Reverse: CCTGCTCATAGTAGTGGCCG	Sigma	

**Software and algorithms**		

Fiji-image J		https://fiji.sc/
Prism 10	Graphpad	https://www.graphpad.com/
Flowjo 10.9.0.	BD	https://www.flowjo.com/previous-versions-flowjo

### Experimental model and study participant details

The use of the outbred Kunming White and C57BL/6 mice was approved by the Animal Advisory Committee at Henan Provincial Hospital and the Institutional Animal Care and Use Committee (IACUC) at the University of Connecticut, respectively. Eight-week-old male Kunming white mice were purchased from Zheng Zhou University and maintained in the animal vivarium of Henan Provincial Hospital. Four-week-old female, and 8-week-old male C57BL/6 mice were purchased from Charles River, and maintained in the animal vivarium at the University of Connecticut. For oocyte retrieval, females were intraperitoneally (IP) injected with 5 IU of Pregnant Mare Serum Gonadotropin (PMSG) (G4877, Sigma, St. Louis, MO, USA) followed by 5 IU human Chorionic Gonadotropin (hCG) (C1063, Sigma, St. Louis, MO, USA) 48 h later. Females were subsequently euthanized 14 h later by cervical dislocation without anesthesia and cumulus-oocyte complexes (COCs) were collected from the oviduct ampulla in 500 μL human tubal fluid (HTF; MR-070, Millipore, Darmstadt, Germany). Sperm for IVF and immunofluorescence were collected by puncturing the caudal epididymis with hypodermic needles in PBS supplemented with 5 mg/mL BSA (A9418, Sigma, St. Louis, MO, USA).

Commercial frozen bovine semen was purchased from Genex (Shawano, WI) and their use in this study was permitted by ST Genetics (Navasota, TX). Frozen human semen was donated by volunteers at the Henan Provincial Hospital with consent.

### Method details

#### Sperm swim-up and R848 treatment

For swim-up and R848 treatment (Figure 1A), sperm were released in 500 μL HTF in 4-well dishes, and incubated for 15 min in 37°C, 5.5% CO_2_. The sperm were then transferred to 1.5 mL Eppendorf tubes with prewarmed R848 (Novus Biologicals, Littleton, CO, USA) in HTF at 2X the treatment concentration. The tubes were vertically positioned in the incubator for 1 h. Three layers of the sperm suspension, the lower layer at 100 μL, and upper and middle layers at 400 μL and 500 μL, respectively, were collected by careful aspiration with blunt pipette tips. These volume assignments by layer were identical to those described by Umehara et al. (2020).^6^ The method presented in Figure 2C used the same preparation method except that the sperm were centrifuged at 37°C in 300g for 5 min before collection. The precipitation at the bottom of the tube was discarded. Sperm in different layers were counted on hemocytometers. The percentage of sperm in each layer was calculated by dividing the number of sperm in that layer by the total sperm count, which was the sum of sperm from all three layers.

#### Immunofluorescence staining of sperm

Sperm were spread on glass slides and air-dried for 30 min at 37°C. They were then fixed in chilled (−20°C) methanol for 5 min at 4°C. Subsequently, the sperm were washed twice with PBS for 10 s each and non-specific binding was blocked by 10% heat inactivated goat serum in PBS supplemented with 0.1% Triton for 2 h. The primary antibodies, dilutions and working concentrations are listed in Table 3. After washing with 0.3% (v/v) Triton X-100 in PBS, sperm were incubated with FITC-conjugated goat anti-rabbit IgG at 1:200 dilution (#ab6717; Abcam, Cambridge, UK) for 1 h at room temperature. After the second antibody incubation, slides were washed 3 times in PBST (0.1% tween) for 5 min each and stained with DAPI (#AR1176; Boster, Wuhan, CN). Slides were mounted in Prolong Gold anti-fading solution (#P10144; Invitrogen; Carlsbad, CA, USA) and covered with coverslips. For negative controls of the stain, the first antibody was replaced with 10% heat inactivated goat serum in PBS supplemented with 0.1% Triton X-100.

**Table 3:** Summary of primary antibodies used

Antibody	Antigen source	Manufacture, catalog #	Procedures and working dilutions
Anti-TLR7	Human	Bioss; bs-6601	Mouse, bovine, human immunofluorescence (1:10); Western blots (1:1000)
Anti-TLR7	Mouse	Abcam; ab24184	Mouse immunofluorescence (25 mg/mL)
Anti-TLR8	Human	Abcam; ab180610	Mouse, bovine immunofluorescence, (1:10); Western blots (1:1000)
Anti-TLR8	Human	Invitrogen; PA5-102413	Human immunofluorescence (1:20)
Anti-acetyl-α tubulin	Human	Invitrogen; PA5-105102	Mouse, bovine, human Immunofluorescence (1:100); Western blots (1:2000)

#### Fluorescence *in situ* hybridization of human sperm (FISH)

Human sperm on slides was first immunofluorescence-stained for TLR7 or 8. For FISH, DNA was denatured at 75°C for 15 min by immersing the slides in the Isothermal denaturation solution (35 mL 100% ethanol +15 mL Isothermal denaturing reagent; #SL20041-0500, Cellay, Cambridge, MA, USA) for 12 min at room temperature. Then the slides were washed in 85% and 100% ethanol for 5 s each. After drying in air, sperm were hybridized with the Oligo Y chromosome FISH Probes (#OF2-0204-0100, Cellay, Cambridge, MA, USA) under coverslips for 7 min at 37°C. Slides were washed in the 2X SSC (saline sodium citrate; #NH0044, Leagene, Beijing, CN) for 5 min and coverslips removed. Slides were then washed with the Isothermal wash solution (400 mL Isothermal denaturing reagent +5 mL 10X SSC in 45 mL ddH_2_O) for 3.5 min and 2X SSC for 5 min. Finally, sperm were mounted in Antifade+DAPI (#SL20013-0001, Cellay, Cambridge, MA, USA) and observed under a coverslip.

#### Sperm DNA extraction and TaqMan real-time PCR

Mouse sperm from the three swim-up layers were washed in the wash buffer (150 mM NaCl, and 10 mM EDTA) and centrifuged at 2500 g for 10 min. After removing the supernatant, the sperm were resuspended in 150 μL lysing buffer (100 mM Tris·Cl; pH 8.0; 10 mM EDTA; 500 mM NaCl; 1% SDS; 2% β-mercaptoethanol) with 50 μL Proteinase K solution (EO0491, Thermo Scientific, Vilnius, Lithuania). The mixture was incubated at 56°C for 4 h and subjected to the extraction procedure of QIAamp DNA Mini Kit (56304, Qiagen, Hidden, Germany). The DNA concentration was determined using Nanodrop™ One.

The ratio of mouse X-to Y-sperm was determined by the absolute quantitative method using FastStart TaqMan® Probe Master (Roche, Mannheim, Germany). The probes and primers for the SRY (sex-determining region Y protein) and XIST (X-inactive specific transcript) genes are shown in Table 4.

**Table 4:** TaqMan real-time PCR primers and probes used for sexing in the mouse

Gene	GenBank ID	Primers/Probes	Sequences	Product lengths	Annealing temperature
SRY	GenBank: X55491.1	probe	GCAGAATCCCAGCATGCAAA	156 bp	60°C
		forward primer	GTGACACTTTAGCCCTCCGA		
		reverse primer	CCACCTGCATCCCAGCTGCTTGC		
XIST	GenBank: L04961.1	probe	GATAGACGAACCAGCTCCCA	114 bp	
		forward primer	CCGCCGAGTACTTAGGTCTT		
		reverse primer	ACTGCGCGCAGCAACATGCA		

To calculate the X- and Y-sperm ratio, first the genome copy number in a DNA sample was calculated from its concentration using the following formula:

$$Mouse\;genome\;copy\;Number=\left(\frac{Amount\;of\;DNA\;\left(g\right)\times 6.022\times {\;10}^{23}\left(copies/mol\right)}{1.5\times 10^{9}\times 660\;\left(g/mol\right)}\;\right)$$

Where the sperm genome is considered to contain 1.5 billion base pairs $\left(10^{9}\right)$, each of which has a molecular mass of 660 g/mol. Then the standard curves for the X and Y chromosomes were generated using the Ct values of real-time PCR from serially diluted mouse genomic DNA samples, ranging from 10 to 10^6^ copies. A simple linear regression method was used to calculate the standard curves (Figure S3). The X- and Y-chromosome copy numbers of a mouse sperm DNA sample were calculated from the standard curve. The percentage of X chromosome was calculated by the following equation:

Xpercentage=X−chromosomecopynumber/(X−chromosomecopynumber+Y−chromosomecopynumber).

A validation experiment was conducted by mixing male and female mouse genomic DNA at ratios of 2:1, 1:1, 1:1.5, and 1:2, generating expected X:Y ratios of 2:1, 3:1, 4:1, 5:1.

#### *In vitro* fertilization and mouse embryo culture

Mouse cumulus-oocyte complexes (COCs) were collected in 500 μL HTF from the oviduct ampulla of superovulated female mice. Mouse sperm from the three swim-up layers were washed in 1 mL HTF to remove R848 and centrifuged at 300g, 37°C for 5 min. One million sperm were added into the droplets of mouse COCs and incubated at 37°C, 5.5%CO_2_, 6% O_2_ for 6 h. The presumptive zygotes were moved to FHM (MR-025-D, Millipore, Darmstadt, Germany) to remove cumulus cells. The denuded embryos were cultured in KSOM (MR-106-D, Millipore, Darmstadt, Germany) at 37°C, 5.5% CO_2_, and 6% O_2_. Cleavage and blastocyst development was observed on Days 2 and 4 (Day 0 = fertilization).

#### Embryo sex determination by PCR

DNA was extracted from individual mouse embryos using the Guide-it Mutation Detection kit (Cat. Nos. 631443, Takara, Kusasu, Japan). PCRs for both SRY (for Y chromosome) and XIST (for X chromosome) were conducted on all samples using DreamTaq Green PCR Master Mix (2X) (K1082, Thermo Scientific, Vilnius, Lithuania). The primer information is shown in Table 5. The PCR products were resolved on 2% agarose gels.

**Table 5:** Primers for PCR of mouse embryo sex determination

Genes	GenBank ID	Primer sequences	Product lengths	Annealing temperature
SRY	GenBank: X55491.1	F’: AAGCGCCCCATGAATGCATT	105 bp	61°C
		R’: TCCCAGCTGCTTGCTGATCT		
XIST	GenBank: NR_001463.3	F’: CAGAGGAAGAGGAAGGCACG	147 bp	
		R: CCTGCTCATAGTAGTGGCCG		

#### Western blotting

Bovine sperm were lysed using the RIPA lysis and extraction buffer (Cat. 89900, Thermo Scientific, US). For TLR7 detection, sperm protein lysis was mixed with 4x NuPAGE LDS Sample Buffer (Cat. NP0007, Invitrogen, Carlsbad, CA, USA) for denaturing gel electrophoresis. For bovine TLR8, the sperm protein lysis was reduced by adding NuPAGE™ Sample Reducing Agent (10X) (Cat. NP0004, Invitrogen, Carlsbad, CA, US). This step was necessary as TLR8 showed poor detectability under non-reducing conditions. Full-length TLR8 protein (∼120 kDa) contains an intermolecular disulfide bond, after reducing, the TLR8 will be cleaved into the 70–80 kDa N-terminal and 50–60 kDA C-terminal fragments.^46^

The protein mixtures were resolved using Mini-PROTEAN TGX Precast Gel (10%) (cat. 4561034, Bio-Rad, USA) electrophoresis and transferred to PVDF membrane (Cat. NO. IPVH00005, Merck Millipore, Carrigtwohill, Co. Cork, IRL). The membranes were blocked in 5% dried milk (#1706404XTU, Bio-Rad, US) in 1X TBST and incubated with primary antibodies overnight at 4°C (Primary antibody listed in Table 3). After washing in TBST 3 times for each 5 min, the blots were incubated with goat anti-rabbit IgG secondary antibody conjugated with HRP (#32460, Invitrogen, USA). After another three washes in TBST, blots were detected by the Enhanced Chemiluminescence (ECL) detection kit (Cat. # 170–5060, Bio-Rad, USA). Alpha tubulin was used as the loading control and probed with anti-acetyl-alpha tubulin (Lys40) polyclonal antibody (#PA5-105102; Invitrogen, USA) after stripping away the antibodies for TLR7 or TLR8. The exposure times for chemiluminescence were 5 min for TLR7 and TLR8 and 16 s for acetyl-α-tubulin. Qualification was conducted using the Fiji ImageJ software.

#### Flow cytometry analysis

Bovine and mouse sperm were fixed in 100% methanol for 5 min at 4°C, washed in PBS, and permeabilized in 0.1% Triton X-100 in PBS for 15 min. They were then blocked in 10% heat-inactivated goat serum in PBS for 1 h at room temperature. Sperm were probed with the corresponding primary antibodies (Table 3) overnight at 4°C. The sperm were washed in PBST 3 times, and stained with DAPI (#62248; Invitrogen, USA). The sperm were analyzed using a BD FACSymphony S6 Cell Sorter.

### Quantification and statistical analysis

Data from three replicates were analyzed by GraphPad Prism 10.1.2 with Student’s t test or one-way ANOVA followed by Tukey’s test. The expected and observed values were compared using One sample t-test (∗*p* ≤ 0.05, significant; ∗∗*p* ≤ 0.01, highly significant). Data were presented as the mean ± SD. Flow cytometry data were analyzed and graphed with Flowjo 10.9.0. The deviations between the observed and expected results of TLR7/8 and FISH stains from a human donor were evaluated using 95% binomial proportion confidence intervals with the formula

$$\left(LB,UB\right)=\hat{p}\pm 1.96\sqrt{\frac{\hat{p}\left(1-\hat{p}\right)}{n}}$$

where $\hat{p}$ is the observed proportion, 1.96 is the 95% *Z* score, and n is the total number of observations, LB and UB are the lower and upper boundaries, respectively.

## Supplemental information

Document S1. Figures S1–S3

Data S1. Agarose gel images for mouse embryo sex determination, related to Figure 1G

Data S2. Western Blot images for TLR7 and 8 in bovine sperm, related Figures 4C–4F

Data S3. Raw data in Excel tables for percentages of sperm in different swim-up layers, related to Figures 1B and 1C; mouse embryo development rates, related to Figures 1E–1G; X:Y sperm ratios after R848 treatments, related to Figures 2A, 2B, and 2D; percentages of positively stained mouse sperm, related to Figures 3B and 3D, percentages of positively stained bovine sperm, related to Figures 4B and 4H; quantification of Western blots, related to Figures 4D–4F; percentages of human sperm stained positively, related to Figures 5B; and percentages of positively stain mouse sperm of C57BL/6, related to Figures S2B and S2C
