# Supplementary material for: Evidence against the role of toll-like receptors 7 and 8 in sex selection in mice, cattle, and humans
Source: iScience. 2025 Jul 18;28(9):113164. doi: 10.1016/j.isci.2025.113164 (PMC12409319; doi:10.1016/j.isci.2025.113164)
Supplement: Document S1. Figures S1–S3 [file mmc1.pdf]

iScience, Volume *28*

Supplemental information

Evidence against the role of toll-like receptors 7 and 8 in sex selection in mice, cattle, and humans

Ruifeng Zhao, Jiaxi Liu, Azizollah Bakhtari, Jianing Shen, Alexa Fayad-Costa, Bin Wang, and Xiuchun Tian

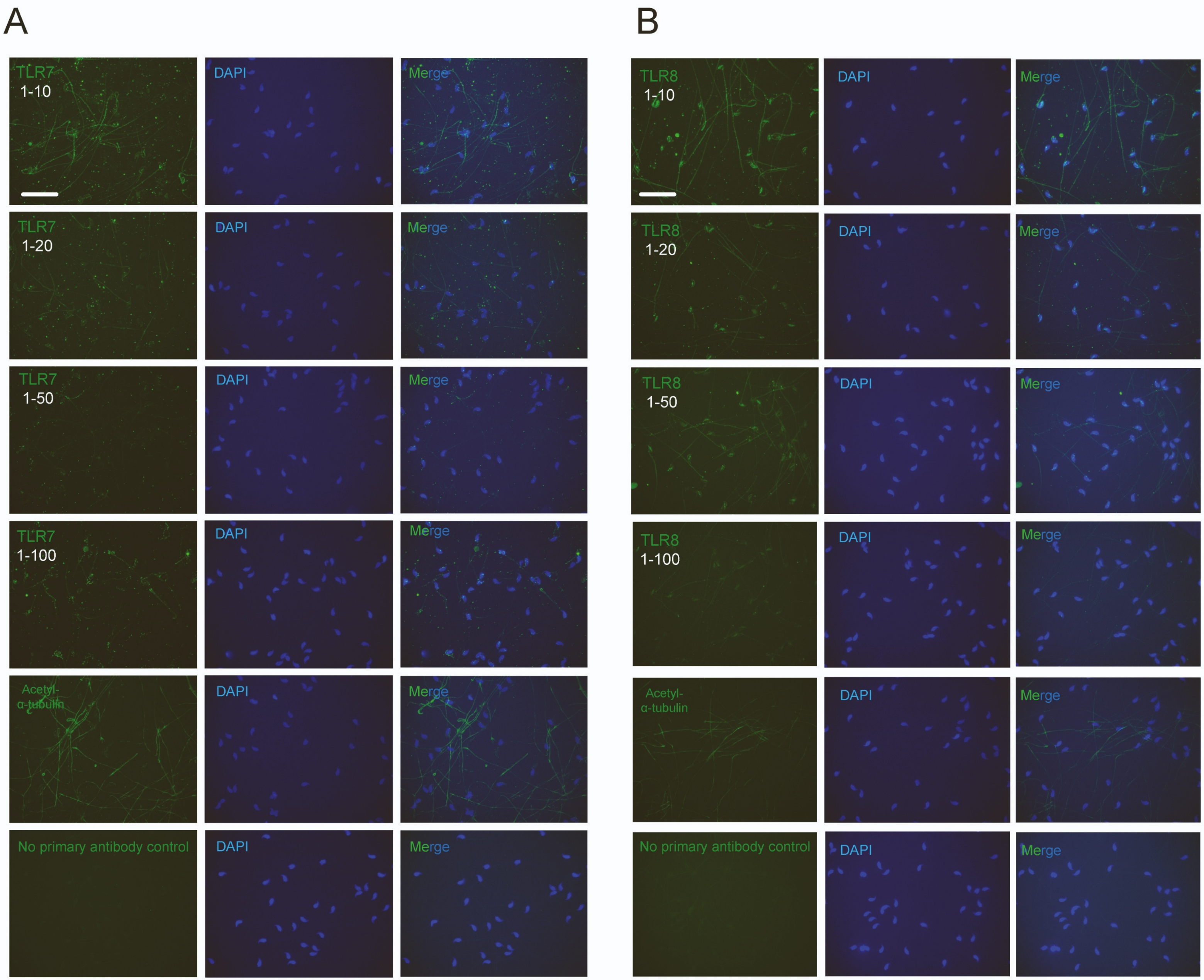

Supplementary Figure S1. Titration of TLR7 (A) and TLR8 (B) primary antibodies for murine sperm immunofluorescence. TLR7/8 stain (green) and DAPI (blue), Scale bar=21 μm.

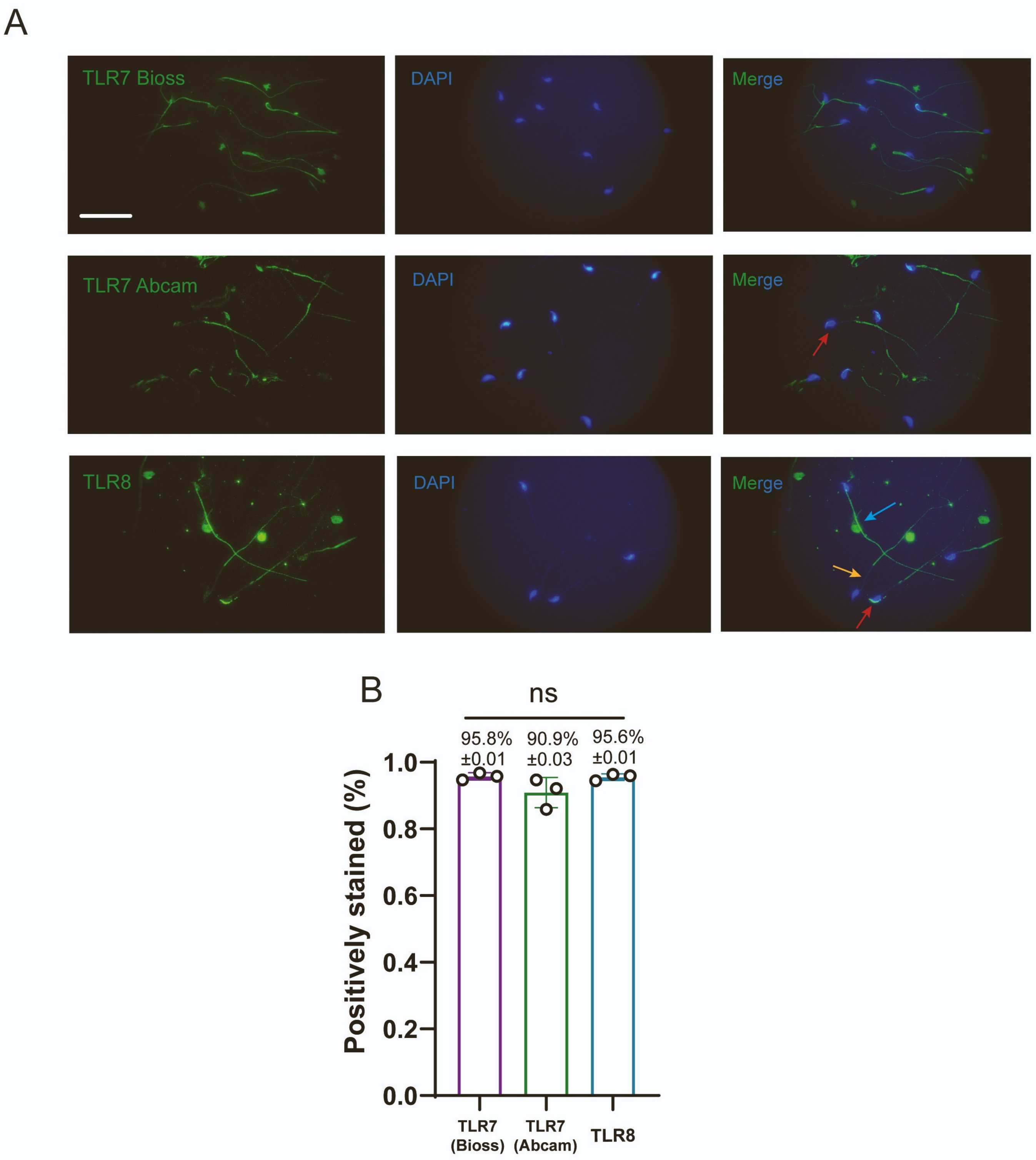

Supplementary Figure S2. Localization of TLR7 and TLR8 in a second mouse strain (C57BL/6). (A) TLR7/8 stain (green) and DAPI (blue); the Abcam antibody against TLR7 stained the lower half of the sperm tail as well as the acrosome of the sperm (red arrow); TLR8 antibody stained either the lower half (yellow arrow) or the entire sperm tail (blue arrow), as well as the acrosome (red arrow), Scale bar=25μm. (B) The percentages of C57BL/6 sperm positively stained for TLR7 and TLR8. The results did not differ from those of Kunming mice shown in Figure 1.

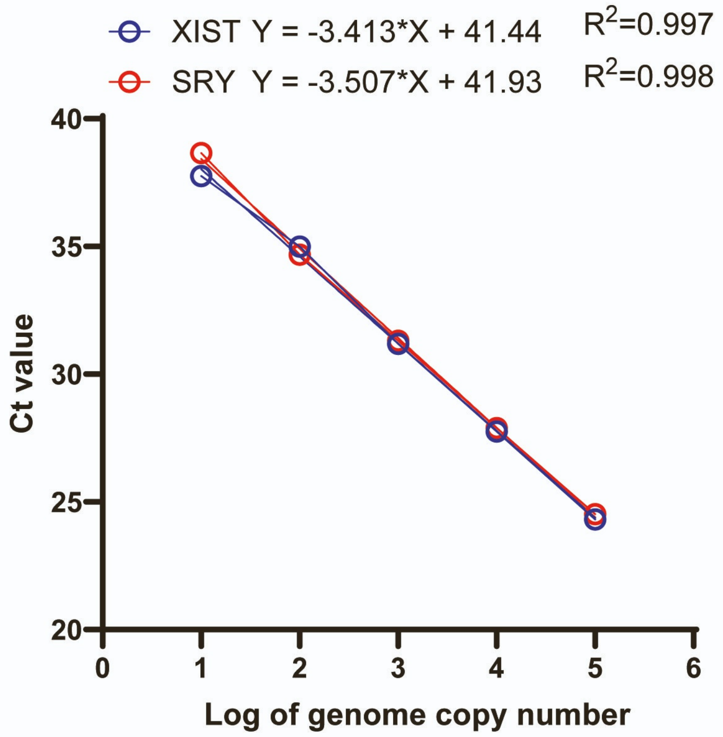

Supplementary Figure S3. A representative standard curve of TaqMan real-time PCRs for XIST and SRY.
