# Supplementary figures and images for: Evidence against the role of toll-like receptors 7 and 8 in sex selection in mice, cattle, and humans
Source: iScience. 2025 Jul 18;28(9):113164. doi: 10.1016/j.isci.2025.113164 (PMC12409319; doi:10.1016/j.isci.2025.113164)

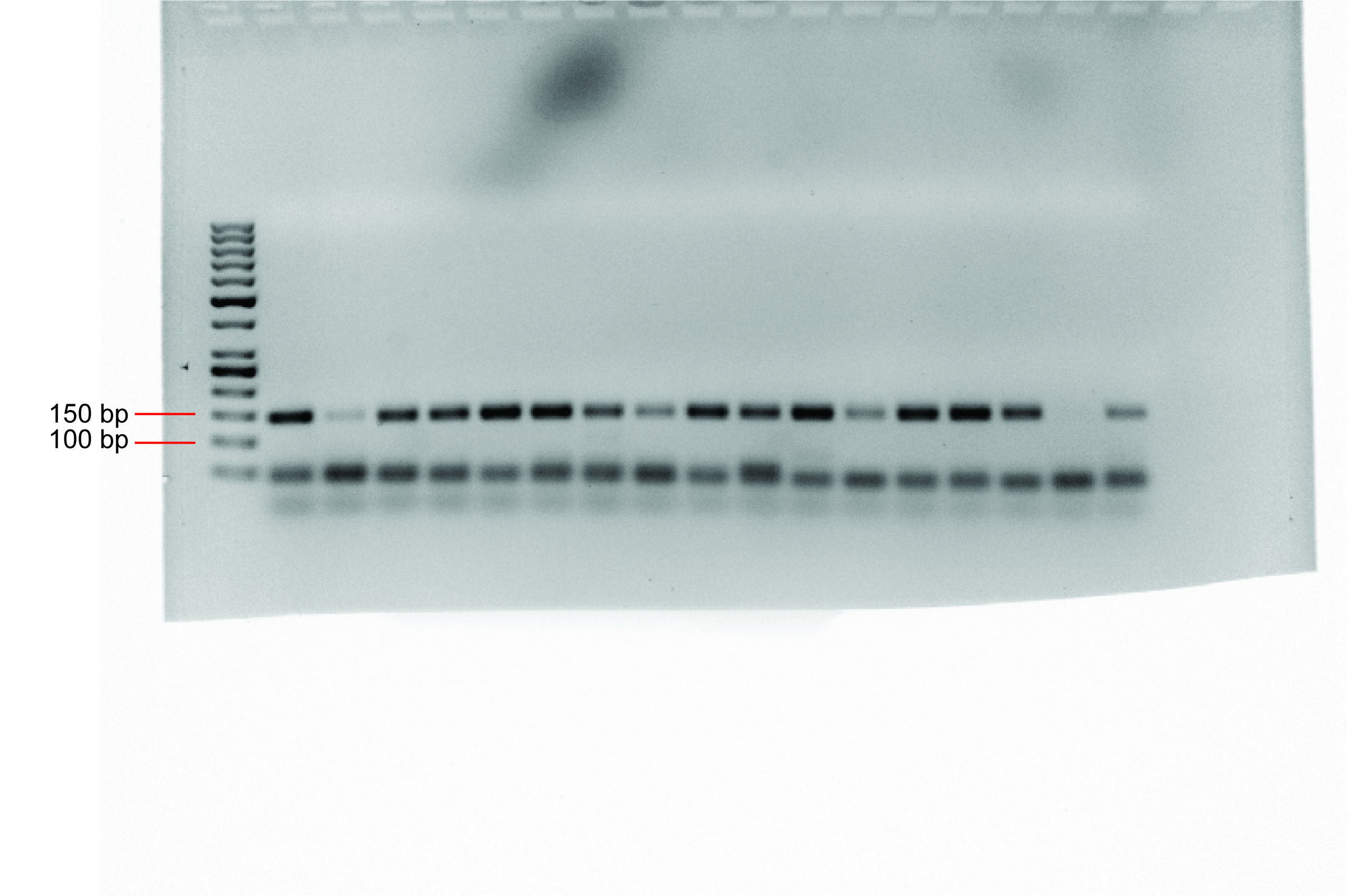

Supplement: Data S1. Agarose gel images for mouse embryo sex determination, related to Figure 1G [file mmc2.zip › Data S1/GEL 7 0.03U13-0.03U22X 0.03L1-0.03L7X 4.20.23.tif]

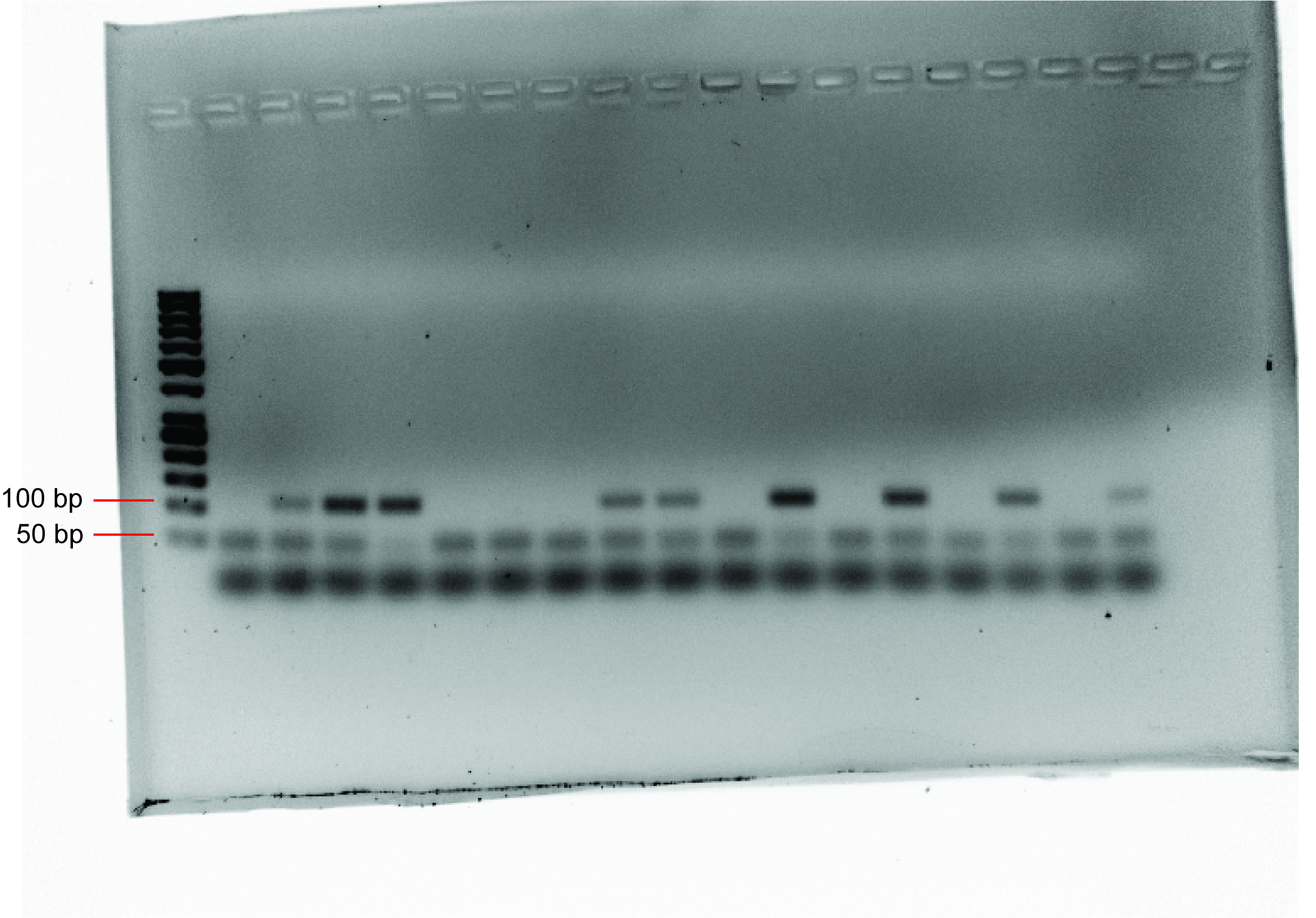

Supplement: Data S1. Agarose gel images for mouse embryo sex determination, related to Figure 1G [file mmc2.zip › Data S1/GEL 8 0.03U13-0.03U22y 0.03L1-0.03L7y 4.20.23.tif]

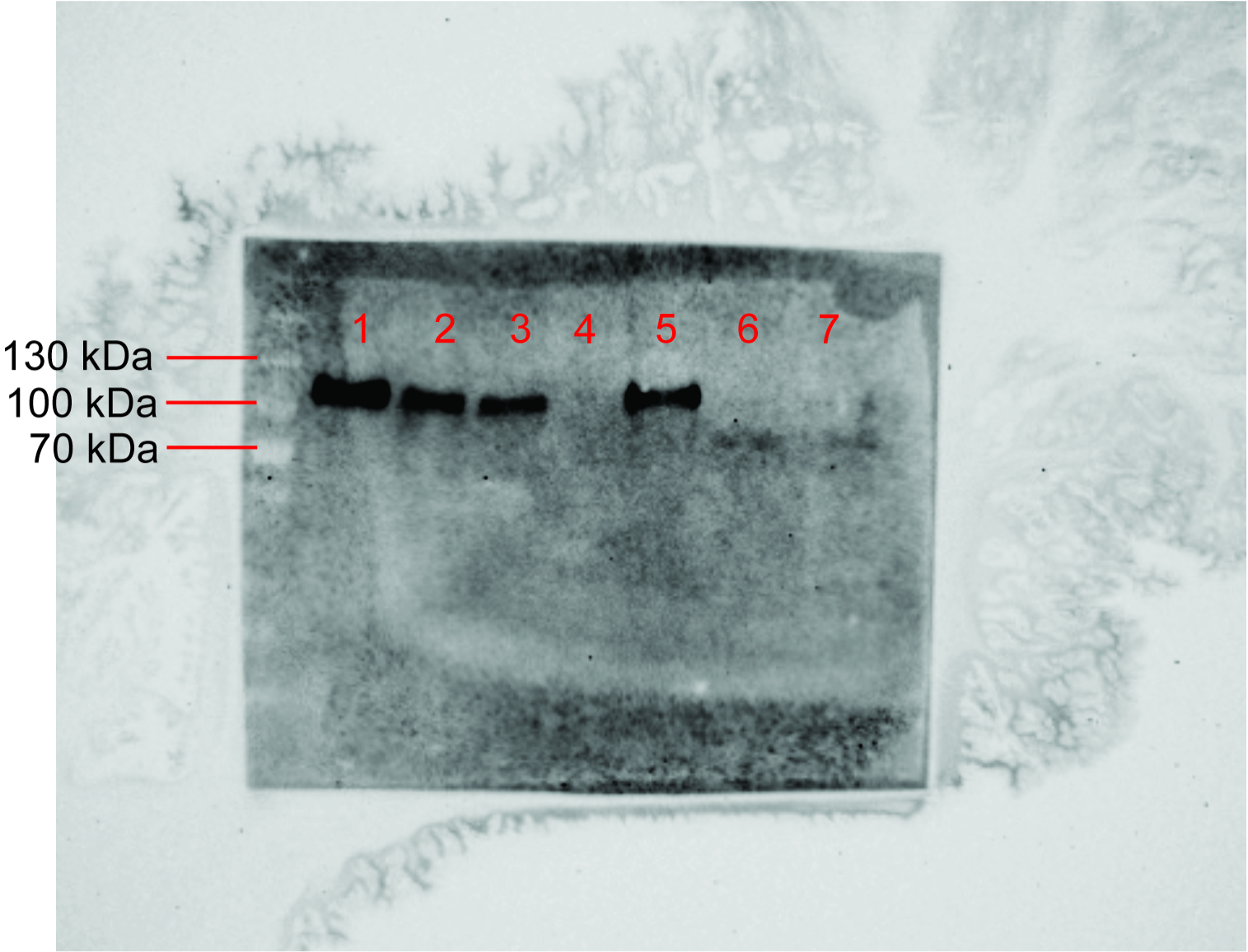

Supplement: Data S2. Western Blot images for TLR7 and 8 in bovine sperm, related Figures 4C–4F [file mmc3.zip › Data S2/TLR7/TLR7.tif]

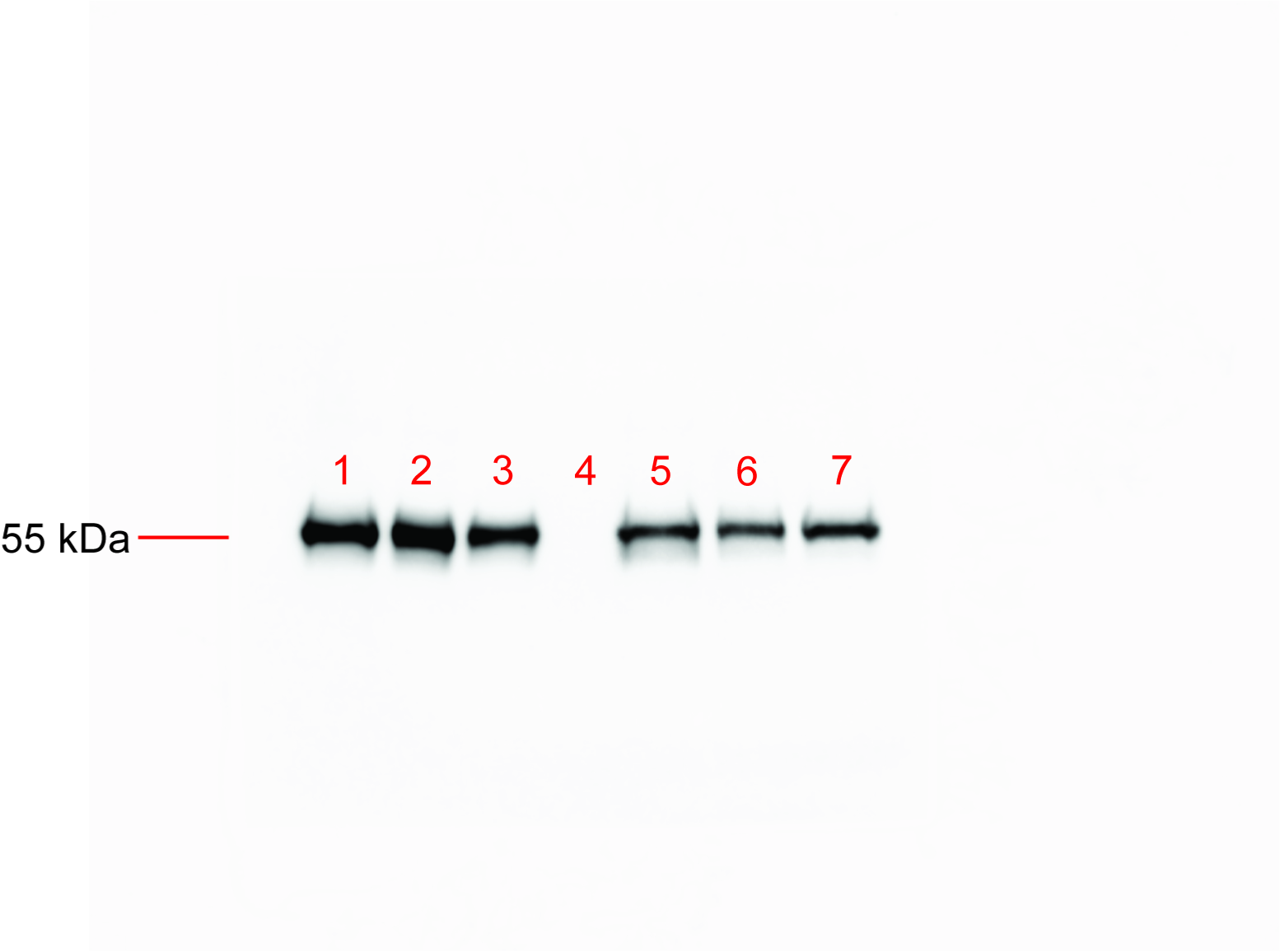

Supplement: Data S2. Western Blot images for TLR7 and 8 in bovine sperm, related Figures 4C–4F [file mmc3.zip › Data S2/TLR7/Tublin after stripping TLR7.tif]

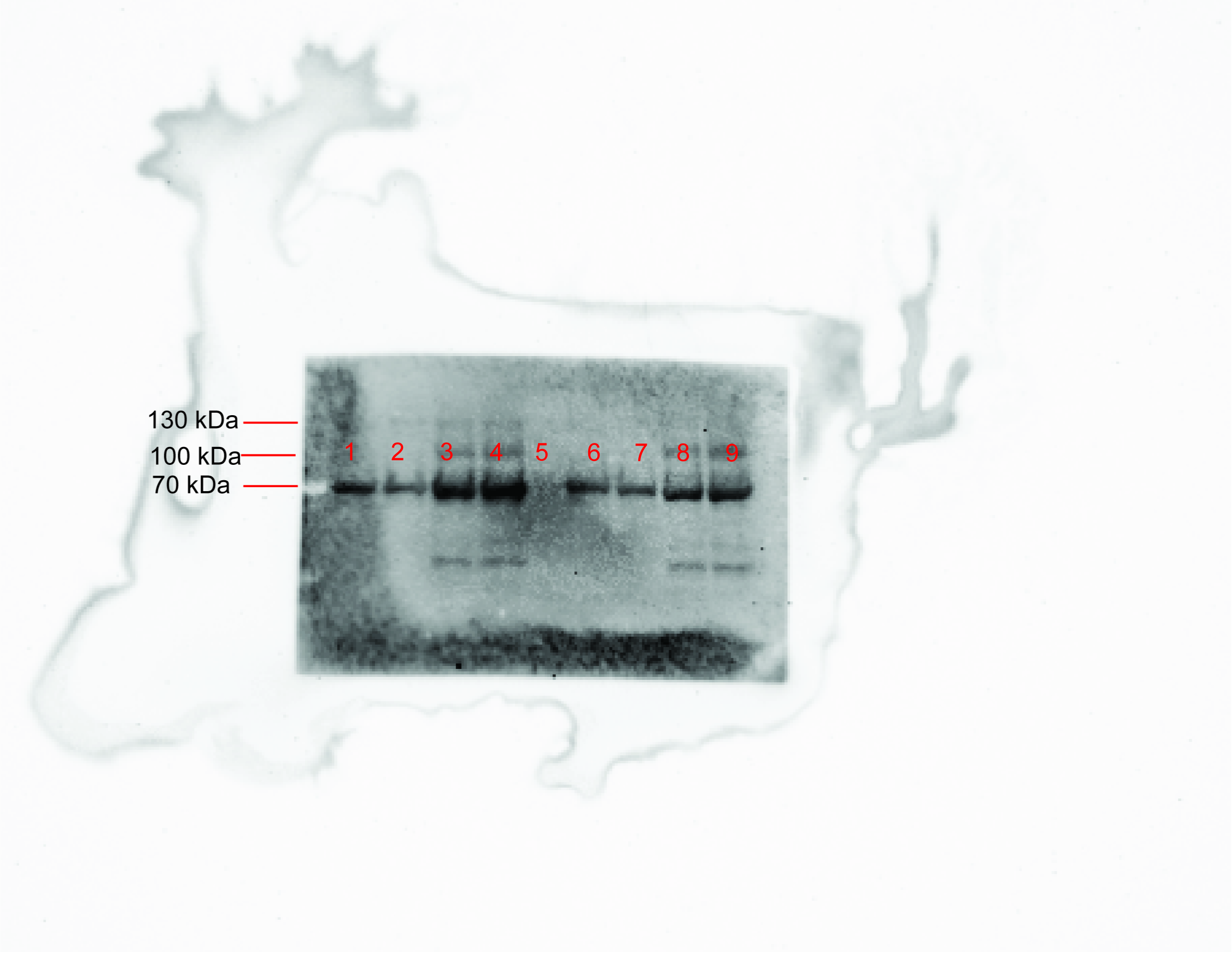

Supplement: Data S2. Western Blot images for TLR7 and 8 in bovine sperm, related Figures 4C–4F [file mmc3.zip › Data S2/TLR8/TLR8.tif]

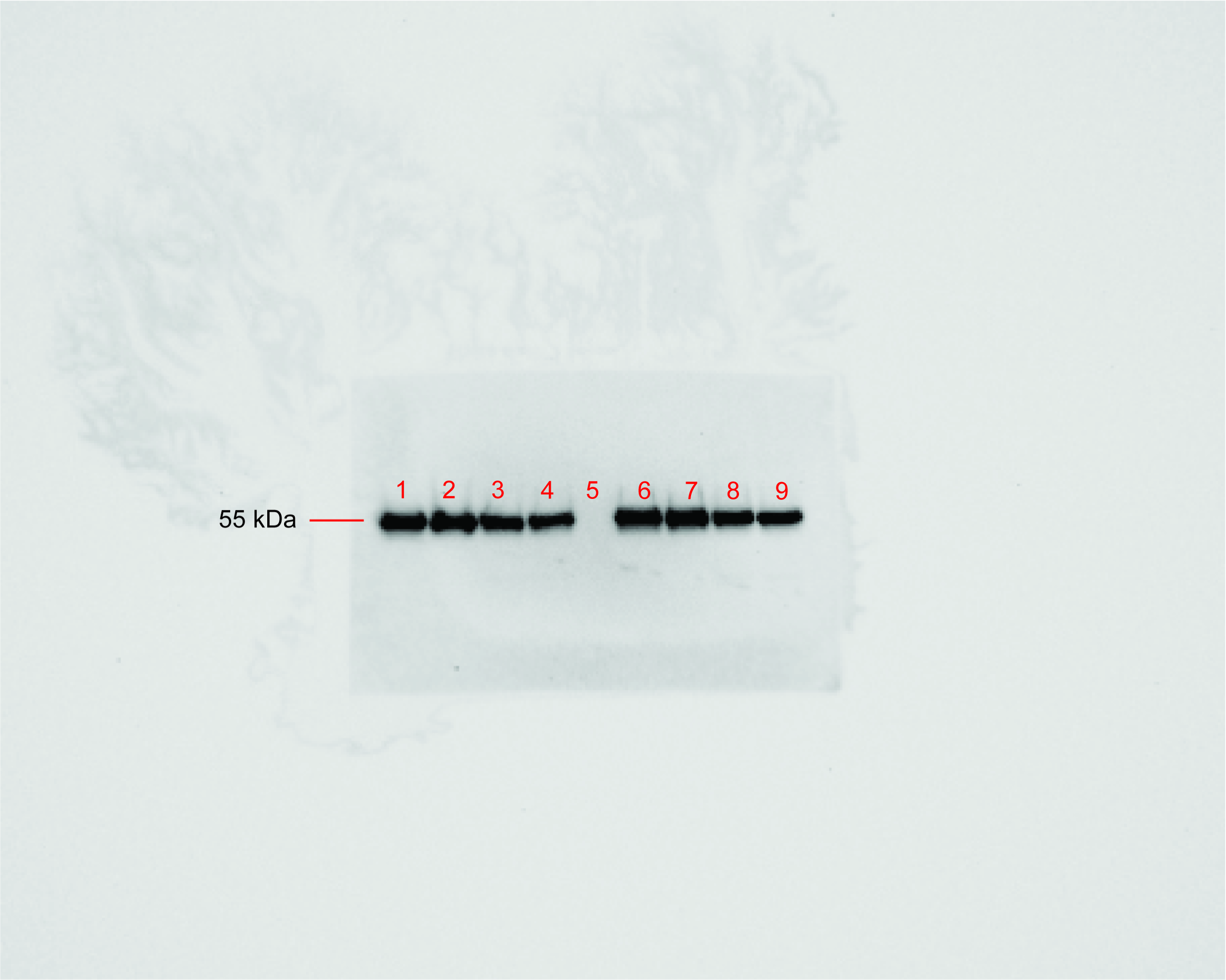

Supplement: Data S2. Western Blot images for TLR7 and 8 in bovine sperm, related Figures 4C–4F [file mmc3.zip › Data S2/TLR8/Tublin after stripping TLR8.tif]
